# Qiviut Trace and Macro Element Profile Reflects Muskox Population Trends

**DOI:** 10.1002/ece3.71020

**Published:** 2025-02-20

**Authors:** Eleanor R. Dickinson, Jesper Bruun Mosbacher, Colleen Arnison, Kimberlee Beckmen, Steeve D. Côté, Juliette Di Francesco, Sophia V. Hansson, Elham Z. Jahromi, David W. Kinniburgh, Gäel Le Roux, Lisa‐Marie Leclerc, Fabien Mavrot, Niels M. Schmidt, Michael J. Suitor, Joëlle Taillon, Matilde Tomaselli, Susan J. Kutz

**Affiliations:** ^1^ Faculty of Veterinary Medicine University of Calgary Calgary Alberta Canada; ^2^ Fram Centre Norwegian Polar Institute Tromsø Norway; ^3^ Parks Canada Inuvik Northwest Territories Canada; ^4^ Alaska Department of Fish and Game Fairbanks Alaska USA; ^5^ Caribou Ungava, Département de Biologie & Centre d'études Nordiques Université Laval Quebec Quebec Canada; ^6^ Faculty of Veterinary Medicine University of Montreal Saint‐Hyacinthe Quebec Canada; ^7^ Centre de Recherche sur la Biodiversité et l'Environnement Université de Toulouse, CNRS UMR 5300, IRD, Toulouse INP, Université Toulouse III–Paul Sabatier (UT3) Toulouse France; ^8^ Department of Ecoscience and Arctic Research Centre Aarhus University Roskilde Denmark; ^9^ Alberta Centre for Toxicology University of Calgary Calgary Alberta Canada; ^10^ Department of Environment Government of Nunavut Kugluktuk Northwest Territories Canada; ^11^ Fish and Wildlife Environment Yukon Whitehorse Yukon Canada; ^12^ Service de la Gestion des Espèces et des Habitats Terrestres Ministère des Forêts, de la Faune et des Parcs Quebec Quebec Canada; ^13^ Polar Knowledge Canada Canadian High Arctic Research Station Cambridge Bay Nunavut Canada

**Keywords:** hair minerals, non‐invasive bio‐monitoring, *Ovibos moschatus*, population trajectory, ungulate, wildlife nutrition

## Abstract

Understanding the drivers influencing ungulate population dynamics is crucial for developing conservation and management strategies to support wildlife health. Trace and macro elements are vital for ungulate growth, reproduction and survival. Thus, the trajectory of wildlife populations may be associated with element imbalances. Element concentrations can be measured in hair, an increasingly recognised bio‐monitoring tool. However, a better understanding of the relevance for wild ungulate population dynamics is needed. This study aimed to assess if element profiles in hair reflected the population trajectory of a keystone Arctic ungulate, muskox *Ovibos moschatus,* and whether benchmarks could be defined for element concentrations to assess population status. We measured qiviut (hair) element concentrations of 11 muskox populations ranging across northern America, including Greenland, and evaluated the association between element concentrations and different population trajectories. Seven trace and macro elements differentiated increasing populations from declining and stable populations using linear discriminant analysis. In general, copper, selenium, iron, manganese and cobalt tended to be at higher concentrations in increasing populations, whereas zinc and calcium were generally at lower concentrations in these populations, though variations were observed among populations. Benchmarks were defined for copper, selenium and iron, indicating populations were more likely to decline below a threshold concentration of these elements (‘limit’) and increase above a threshold concentration (‘target’). ‘Limit’ benchmarks were defined for zinc and calcium where populations were more likely to be increasing below this threshold value. Hair element profiles are a useful indicator of population trajectory in wild ungulate populations. Identified benchmarks can be used to assess population status, complementing ongoing but irregular and expensive monitoring efforts like population surveys, while trace element concentrations can provide insights into the mechanisms driving population change. Hair samples can easily be collected non‐invasively or alongside other monitoring activities, enhancing proactive wildlife management and conservation.

## Graphical abstract

This study assesses the concentration of trace elements in muskox qiviut (hair) in populations with different trajectories. We found that certain elements, including copper, selenium and iron, were more likely to be higher in increasing populations and lower in declining populations and we defined thresholds for assessing the status of muskox populations.

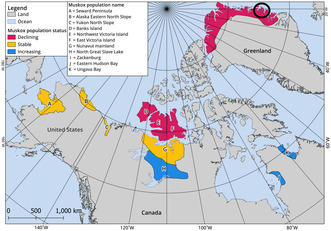

## 1 Introduction

Ungulates are broadly recognised for their role in ecosystem structure and function (Bauer and Hoye 2014; Trouwborst 2019) and hold significant importance for people, especially those that rely on them for food and culture (Tomaselli et al. 2018; Pascual‐Rico et al. 2021). Understanding the drivers of ungulate populations is crucial for informing conservation and management strategies to ensure the persistence of healthy populations (Carpio et al. 2021; Desforges et al. 2021; Kauffman et al. 2021). The reproduction and survival of ungulates are closely linked to nutritional resource availability (Gaillard et al. 2000; Illius and O'Connor 2000). In addition to energy and protein, these resources also include nutrients, such as elements and vitamins, which are associated with health and fitness (O'Hara et al. 2001; Webster et al. 2021). Essential elements that are required for normal physiological functions, such as immunity, reproduction and growth, include trace elements that are required in small amounts (e.g., copper Cu and selenium Se) and macro elements that are required in larger amounts (e.g., calcium Ca and magnesium Mg; Nieder et al. 2018; Underwood 2012; Webster et al. 2021). Imbalances of these elements, either deficiencies or toxicities, can impact ungulates, such as being linked to reduced fertility, poor calf survival, impaired growth and increased adult mortality (Hidiroglou 1979; O'Hara et al. 2001; Soetan et al. 2010).

Deficiencies of trace and macro elements, particularly Cu and Se, have been linked to poor reproductive success and increased susceptibility to infectious diseases in wild ungulates (Flynn et al. 1977; Scaletti et al. 2003; Flueck et al. 2012; Afema et al. 2017; Durkalec et al. 2018). Conversely, elevated levels of elements, including heavy metals, can lead to toxicity, which can also impair immunity and reproduction and thus population productivity (Gamberg et al. 2016; Durkalec et al. 2018). Elements may also interact with each other; for example, molybdenum (Mo) can reduce the bioavailability of Cu (Puls 1994), and Cu deficiency has been linked to high levels of iron (Fe), calcium (Ca) and zinc (Zn; Herdt and Hoff 2011). While the importance of trace and macro elements for growth, reproduction and productivity in domestic livestock is well understood, our understanding of the role of these elements in wild ungulate populations is still limited (Blakley et al. 2000; French et al. 2017; Rioux et al. 2022).

Wild ungulates obtain elements through vegetation and water intake or through alternative sources with higher concentrations of elements, such as natural mineral licks (Klein and Thing 1989; Ayotte et al. 2006; Oster et al. 2018). The quality, abundance and access to elements vary temporally and spatially across heterogeneous geochemical landscapes, impacting the availability of important nutrients to ungulates (Oster et al. 2018; van Beest et al. 2023). This is especially important during periods that require additional resources, such as when females are pregnant or lactating (Tajchman et al. 2018). In areas with distinct growing seasons, ungulate reproduction is often synchronized with the peak of resource availability to exploit high‐quality forage (Couriot et al. 2023). Changes in climate are driving shifts in vegetation diversity, composition and phenology, which can change the availability and uptake of nutrients (Oster et al. 2018). However, few studies have addressed how the availability of elements in natural environments may impact individual or population‐level performance (but see van Beest et al. 2023). Understanding how the uptake of trace and macro elements available in the environment reflects population demographics would allow them to serve not only as a useful bioindicator but also to help us understand the links between the bioavailability of elements, ungulate nutrition and population trends.

The bioavailability of elements is likely to change with shifts in vegetation quality and availability due to climate change (Schmidt et al. 2002). These changes will be pronounced for Arctic ungulates, including caribou *Rangifer* spp. and muskoxen *Ovibos moschatus* because of the rapid rate of climate change in this region (Forchhammer et al. 2002; Mallory and Boyce 2018; Desforges et al. 2021). The energy and protein requirements for these species are generally well understood (Gustine et al. 2011; Barboza et al. 2018; Desforges et al. 2019). However, a better understanding of the dynamics of elements and their impact on ungulates is needed (Blakley et al. 2000; Flueck et al. 2012). Element concentrations have been associated with adult survival in caribou (Rioux et al. 2022) and moose *Alces alces* (O'Hara et al. 2001), and with calf recruitment in muskoxen (Mosbacher et al. 2022; van Beest et al. 2023). While measuring element concentrations in storage organs, such as the liver or kidney, is most common because these organs reflect the availability of elements in the body, hair is being increasingly used as a non‐invasive indicator of element concentration (Jutha et al. 2022). Elements are incorporated into hair during growth, which occurs during a defined period, after which it is separated from the body's metabolism; thus, element status in hair would represent element status in the body during hair growth (Combs 1987). Hair has been used for assessing individual health in woodland caribou *R. t. caribou* (Jutha et al. 2022), and as an indicator for a demographic measure, calf recruitment, in muskoxen (Mosbacher et al. 2022). These findings suggest that certain elements, Cu, Se and Mo, are linked to successful reproduction in muskoxen, which may have important consequences for population trajectories (Mosbacher et al. 2022). While these studies provide promising examples of using hair as an indicator of trace element status, it is important to validate this approach. Mineral status in the body may not always be accurately reflected in hair, and non‐dietary factors such as age and sex can also influence element levels (Combs 1987). However, such methods have the potential to be applied in contexts where traditional monitoring is challenging.

Muskox population monitoring is hindered by limited information due to sparse population surveys, which occur roughly every 10 years due to the significant costs of conducting aerial surveys in the Arctic (Cuyler et al. 2020). Therefore, other monitoring approaches, such as non‐invasive bio‐monitoring tools, are especially valuable. In this study, we focused on understanding the association between element concentrations (Cu, Se, cobalt Co, Zn, manganese Mn, Mg, sodium Na, Fe, Ca, Mo and chromium Cr) and muskox population trajectories based on population surveys. We first explored the association between trace elements measured in muskox qiviut, the dense undercoat hair that is shed annually and drivers of variation such as animal age class, qiviut growth year and sample collection type. Secondly, we examined the association between elements and population trends in 11 discrete muskox populations that were either decreasing, stable or increasing. The final goal was to provide criteria or ‘benchmarks’ to assess muskox population status based on the qiviut element profile.

## 2 Materials and Methods

### 2.1 Study Area and Sample Collection

**FIGURE 1:**
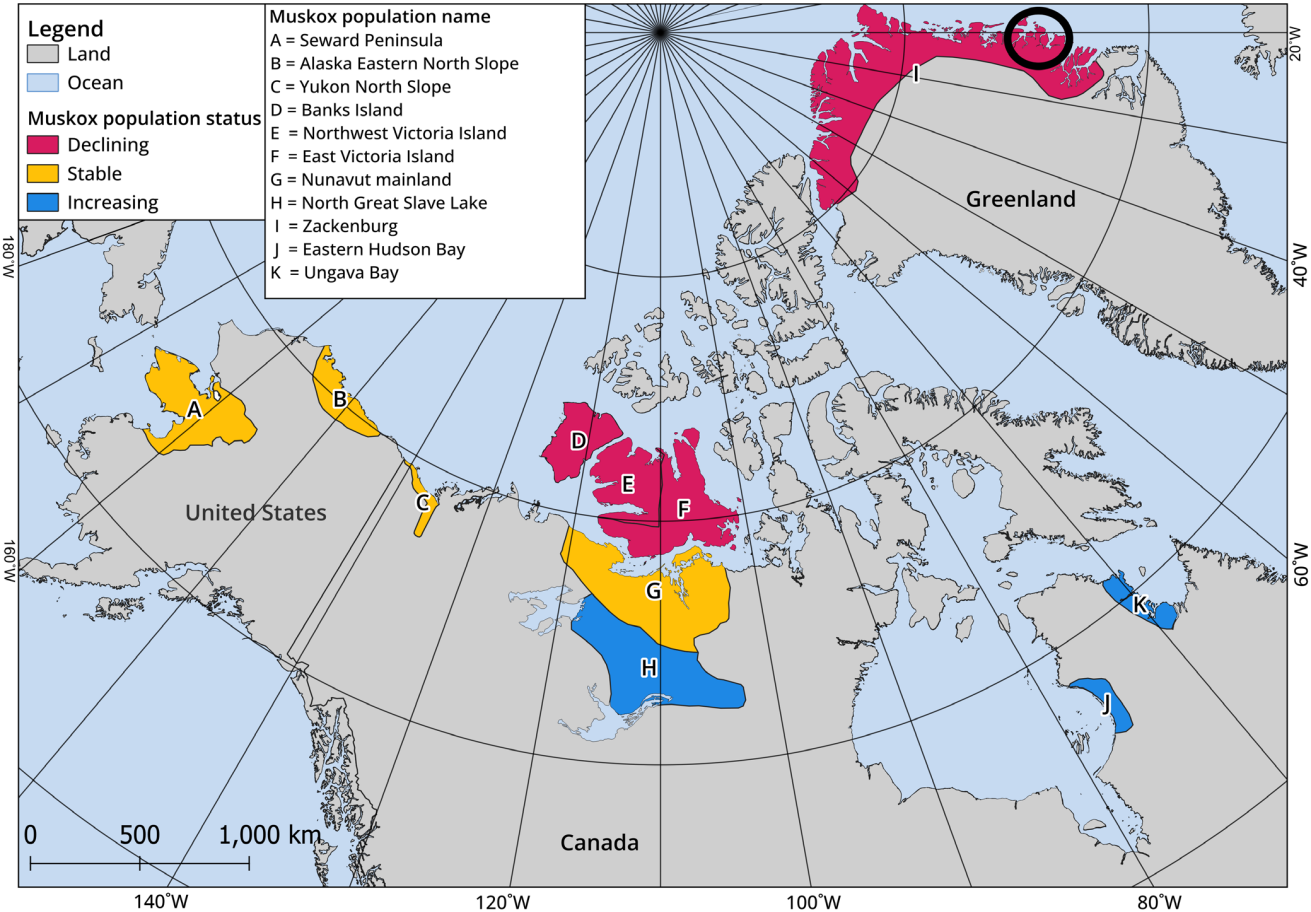
The population or management units from which muskox qiviut samples were collected for this study. The population trend for each population at the time of sample collection is indicated by the colour. The areas were outlined by Cuyler et al. (2020). Samples from Greenland were collected within the black circle, and do not represent the entire range in North Greenland.

We collected qiviut samples from 11 muskox populations with contrasting population sizes and trends, across multiple years and from a large geographical range (Table 1; Figure 1). Of these, 4 populations were increasing, 4 were stable and 3 were declining at the time of qiviut growth as estimated by aerial surveys (Table 1). Muskoxen generally inhabit tundra, shrub tundra and shrubland, and forage in graminoid‐ and *Salix*‐dominated areas (Brodeur et al. 2023). Muskoxen have two types of hair: long continuously growing guard hairs and a dense undercoat called ‘qiviut’ which grows seasonally. Muskox qiviut grows during a discrete time period from spring to autumn (approximately a 6‐month period) and is shed in synchronised moults the following spring–summer (Flood et al. 1989). This means that qiviut can be used to track inter‐annual fluctuations of mineral levels in muskoxen. The qiviut growth period was defined as April to November in the year prior to collection (Di Francesco et al. 2022). In total, we collected 414 qiviut samples from different sex and age (calf, yearling and adult) classes spanning the qiviut growth years 2003–2018 (Table 1). Qiviut samples were collected outside the qiviut growth period using three collection methods. Freshly shed qiviut samples were collected from the ground from individual clumps during the summer months (*n* = 37, as described by Mosbacher et al. 2022). Shaved qiviut samples were collected from the rump of randomly selected tranquilised muskox during muskox captures for radio or GPS collaring (*n* = 143, as described Schmidt et al. 2016) and shaved qiviut samples were collected from the rump of hunted animals (*n* = 234, as described by Di Francesco et al. 2022). Animals were hunted by subsistence and sport hunters in the Canadian Arctic in collaboration with the local Hunters and Trappers organisations near the communities of Ulukhaktok in the Northwest Territories and Kugluktuk and Ekaluktutiak/Cambridge Bay in Nunavut. These are regular activities in the communities, and no animals were killed specifically for this study. Samples were obtained under animal care and wildlife research permits (Appendix S1).

**TABLE 1:** Overview of populations included in this study ranging from Alaska to Greenland with the hair growth years when qiviut element concentration was measured, sample size and collection method. The population trends and sizes from aerial surveys (except Zackenberg which was estimated from ground surveys) relevant to the time of hair formation are reported, including the survey year, the annual change in population size (% change; percent of total population estimate or count), number of animals counted (count) and the estimated population size (estimate), including the 95% confidence intervals (95% CI). The hair growth year refers to the year of hair formation, rather than the year of collection.

Population	Hair growth year(s)	*N*	Collection method	Population trend	Survey year	% change	Count	Estimate	95% CI
Alaska									
Alaska Eastern North Slope^a^	2006–2007	20	Capture	Stable	2015	−1	198		
Seward Peninsula^b^	2008, 2018	16	Capture	Stable	2017	1		2353	1908–2936
Yukon									
Yukon North Slope^a^	2016–2018	20	Capture (*n* = 14); Shed (*n* = 6)	Increasing	2018	8	344		
Northwest Territories									
Banks Island^c^	2017	9	Shed	Declining	2019	−6	1877	10,979	9531–12,427
Northwest Victoria Island^d^	2016–2018	60	Hunted	Declining	2019	−41	2444	5550	3878–7222
North Great Slave^e^	2017	22	Shed	Increasing	2018	11	1656	8098	
Nunavut									
East Victoria Island (MX‐07)^f^	2014–2018	61	Hunted	Declining	2013–2014	−14	1296	10,026	8842–11,210
Nunavut Mainland (MX‐09)^g^	2013–2018	113	Hunted	Stable	2018	−1	87	539	245–833
Quebec									
Eastern Hudson Bay^h^	2016	30	Capture	Increasing	2020	15		2500	1375–3325
Ungava Bay^h^	2018	19	Capture	Increasing	2019	26		4500	3555–5715
Greenland									
Zackenberg^i^	2013, 2015, 2017	44	Capture	Declining	2017	20	25		

^a^ Cuyler et al. (2020).

^b^ Alaska Department of Fish and Game (2017).

^c^ Davison and Baryluk (2021).

^d^ Davison and Williams (2022).

^e^ Cluff et al. (2019) [unpublished].

^f^ Leclerc (2015).

^g^ Leclerc (2018).

^h^ Brodeur et al. (2023).

^i^ Schmidt et al. (2016).

### 2.2 Qiviut Processing and Analyses

All samples were processed at the Alberta Centre for Toxicology, except for the samples from Eastern Hudson Bay, Yukon North Slope (captures) and Zackenberg, which were processed at the Observatoire Midi‐Pyrénées (OMP) using similar protocols and methods (for details see Appendix S1). For each sample, the qiviut was separated from the long, coarse guard hairs and visible contaminants, such as dirt, vegetation and dandruff, were removed with plastic tweezers. The qiviut samples (30 to 50 mg) were then individually washed twice in 95% ethanol and type 1 ultrapure water to remove remaining external contaminants and the lipid layer surrounding the hair, and then oven dried. A subsample of the dried qiviut sample was then digested with 2 mL concentrated nitric acid. The samples were further diluted with ultrapure water before analyses. The qiviut concentrations of 11 elements (Na, Mg, Ca, Cr, Mn, Fe Co, Cu, Zn, Se and Mo) were determined using inductively coupled plasma mass spectrometry (ICP‐MS; 8800 Triple Quadrupole ICP‐MS, Agilent). Each batch of samples included certified reference materials for quality control (NIST 2976 freeze‐dried mussel tissue and DORM‐3 fish protein), a blank sample as a negative control and a randomly selected duplicated qiviut sample. The acceptable criteria of the concentrations of elements measured in reference materials were within ±20% of the certified values, and blanks were negligible for all samples (Tables S1 and S2). Results below the limit of quantitation (LOQ) for certain elements were included in further analysis by assigning values as half the LOQ following Roug et al. (2015). If more than 50% of samples were below the LOQ for a specific element, that element would not be included in the analyses.

### 2.3 Statistical Analyses

All analyses were conducted using R software version 4.3.2 (R Core Team 2023). Six samples were excluded from the analysis due to missing information. Samples with outliers which were more than three times larger than the standard deviation were removed from the analyses (*n* = 6, Sullivan et al. 2021). Generalised linear mixed effects models were used to test the effect of collection method (harvest, capture and ground collection), animal age class (calf, yearling and adult) and sex (male and female) on the concentration of the 11 elements measured using the *lme4* package (Bates et al. 2015). Population location and sampling year were included as a nested random effect. Age class and sex of individuals were not known for all populations (i.e., when samples were collected from the ground), and only samples with known age and sex were included in this analysis (*n* = 259). Model residuals were checked for normality using histograms of residuals and Q‐Q plots, and non‐normal variables were log‐transformed.

A multivariate analysis of variance (MANOVA) was used to assess which qiviut elements were associated with population trend (declining, stable or increasing). A Linear Discriminant Analysis (LDA) was conducted with the package *mass* (Venables and Ripley 2002) to assign these qiviut elements into specific dimensions defined by population trend, and model fit was assessed using the Wilks lambda criterion (Todorov 2007). To assess the model accuracy, leave‐one‐out cross‐validation was used to estimate prediction error, model accuracy, sensitivity and specificity. Cutoffs, which are referred to as benchmarks, were estimated as the midway point between the mean linear discriminant function for each population trend; the lower benchmark was defined between stable and declining populations, and the upper benchmark was defined between stable and increasing populations.

To provide criteria to assess muskox population status from qiviut element concentrations, an ordinal logistic regression was used with the significant elements. Elements were scaled by subtracting the mean and dividing by the standard deviation (Becker et al. 1988). Using predicted probabilities from the ordinal logistic regression, which are the probabilities at which one category becomes more likely than the adjacent category, benchmarks were defined. The lower value or ‘limit’ benchmark was defined as the element concentration below which the population has a higher probability of decline, and the higher value or ‘target’ benchmark was defined as the element concentration above which the population has a higher probability of increase, following benchmarks defined by Peacock et al. (2020).

## 3 Results

A total of 402 samples were included from 11 different muskox populations across multiple years, and all 11 elements were included in the analyses (Table S3). Some variation in hair element concentration was explained by the fixed effects in the models: collection method (Marginal *R* ^2^ (*R* ^2^m) for Na = 0.046; Mg = 0.066; Mn = 0.16; Fe = 0.059; Co = 0.17; Se = 0.28; Mo = 0.052), age (*R* ^2^m for Co = 0.018), sex (*R* ^2^m for Mo = 0.028), and age and sex (*R* ^2^m for Se = 0.032). However, most of the variation was accounted for by the random effect of population and sampling year. Variation explained for the full model including the random effect (*R* ^2^c) ranged from less than 4% for Zn (*R* ^2^c for collection type = 0.038; age and sex = 0.037) to more than 60% variance explained for Se (*R* ^2^c for collection type = 0.64; age and sex = 0.64; Tables S4 and S5).

### 3.1 Linking Element Status to Population Trends

**FIGURE 2:**
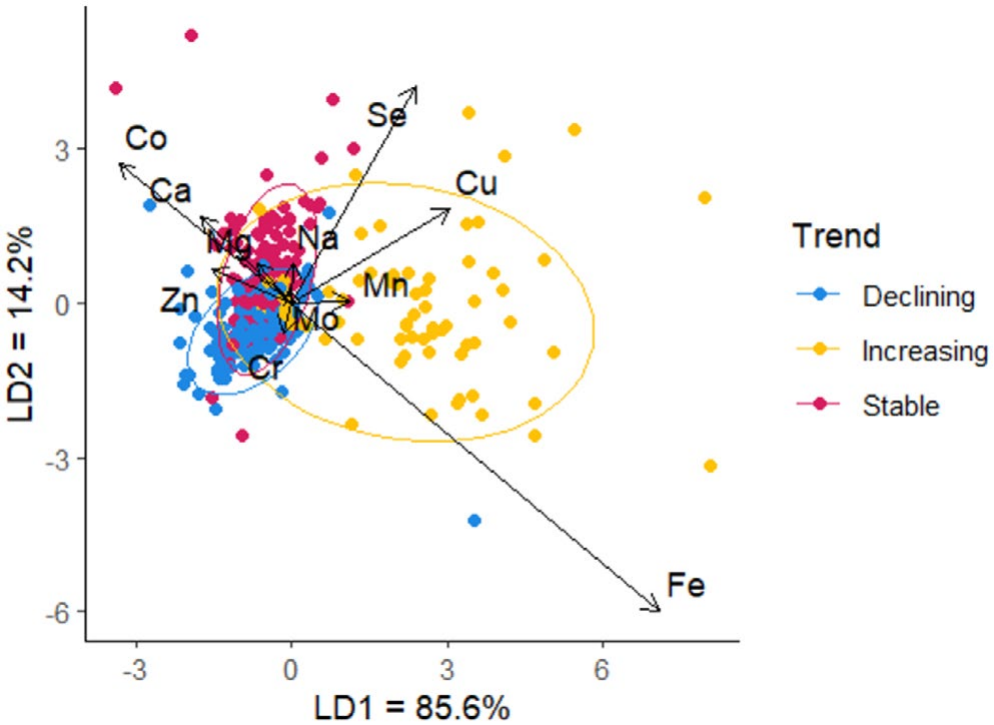
The output of the linear discriminant analysis (LDA) shown as a biplot of the first linear discriminant (LD1) and the second linear discriminant (LD2). The arrows represent the direction and magnitude of coefficients for each element concentration measured in qiviut that was retained in the LDA.

**FIGURE 3:**
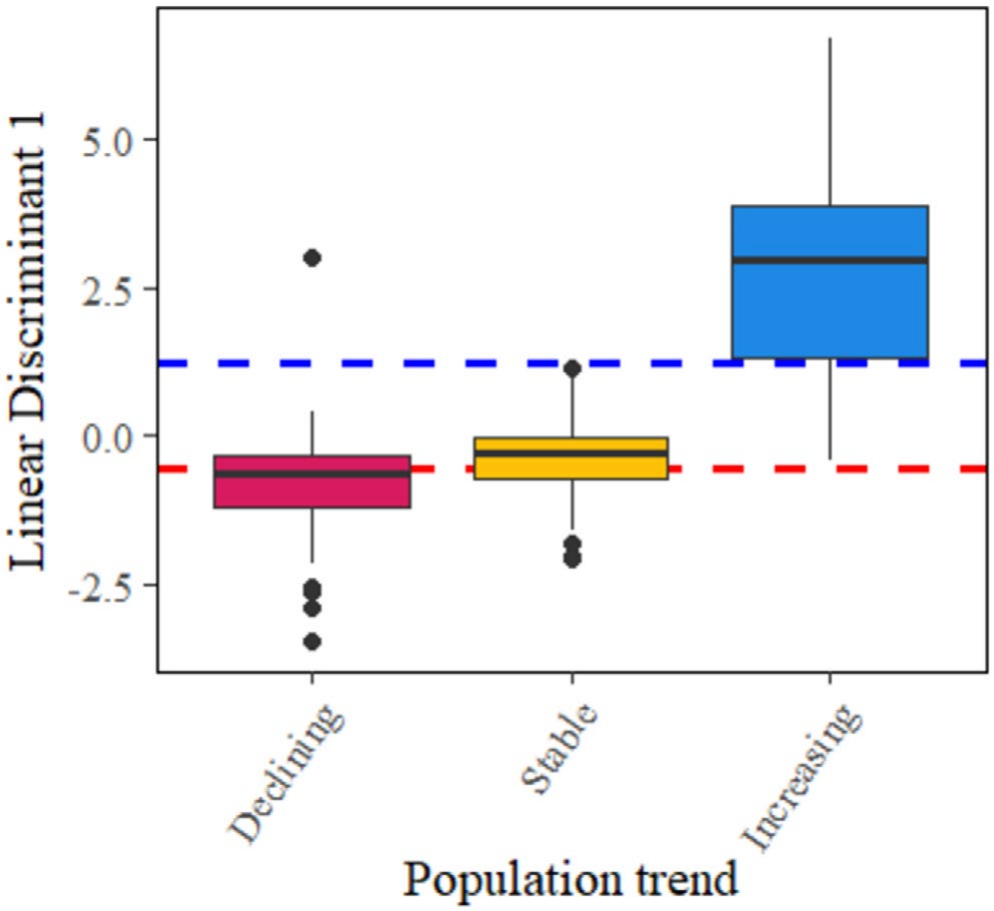
The median linear discriminant (LD1) value, interquartile range, range and outliers for each population trend for muskox populations. The blue line shows the upper benchmark for the first linear discriminant function and the red line shows the lower benchmark for the first linear discriminant function.

Element concentrations differed according to population trend, and the MANOVA indicated that seven elements significantly explained population trend: Cu, Se, Co, Zn, Mn, Fe and Ca (*F* = 44.53, df = 14, *p* < 0.0001; Table 2). Linear discriminant analysis (LDA) indicated two linear discriminant functions explaining 100% of the variation in the data (Wilks' *λ* = 0.27, df = 12, *p* < 0.0001; Figure 2). The first linear discriminant differentiated increasing populations from stable and decreasing populations, explaining 79.5% of the variation. The second linear discriminant, while explaining 20.5% of the variation, differentiated stable from declining populations, and both overlapped with increasing populations. Cross‐validation showed that the LDA had a low error rate and high accuracy when predicting population trend (adjusted error rate = 0.27, sensitivity = 0.91, specificity = 0.76). The ‘target’ benchmark for the first linear discriminant was 1.24, and the ‘limit’ benchmark was −0.56 (Figure 3).

**TABLE 2:** The difference in element concentration between muskox populations with different population trends. A MANOVA (*F* statistic) was used to determine which elements differed among population trends, and the mean concentration of each element (µg/g) with 95% confidence intervals (CI) is shown for each population trend.

Element	df	*F*	*p*	Trend	Mean (µg/g)	95% CI	
						Lower	Upper
Sodium	2	1.77	0.17	Increasing	57.97	41.49	74.45
				Stable	47.24	35.27	59.21
				Declining	50.17	40.51	59.84
Magnesium	2	3.29	0.038	Increasing	119.61	92.60	146.62
				Stable	114.07	95.55	132.59
				Declining	112.79	98.75	126.83
Calcium	2	27.21	< 0.001	Increasing	356.68	325.88	387.47
				Stable	470.78	449.33	492.24
				Declining	418.31	393.28	443.33
Chromium	2	1.29	0.28	Increasing	0.53	0.36	0.70
				Stable	0.24	0.03	0.45
				Declining	0.64	0.12	1.16
Manganese	2	5.93	0.0029	Increasing	5.95	4.36	7.54
				Stable	2.77	1.74	3.80
				Declining	1.65	1.11	2.18
Iron	2	112.98	< 0.001	Increasing	312.94	253.50	372.39
				Stable	20.43	17.57	23.29
				Declining	46.79	32.11	61.46
Cobalt	2	23.45	< 0.001	Increasing	0.11	0.08	0.13
				Stable	0.02	0.02	0.02
				Declining	0.02	0.01	0.03
Copper	2	82.68	< 0.001	Increasing	5.90	5.60	6.20
				Stable	5.26	5.16	5.36
				Declining	4.51	4.38	4.63
Zinc	2	9.33	< 0.001	Increasing	92.50	86.24	98.77
				Stable	104.20	102.40	105.99
				Declining	99.18	97.36	101.00
Selenium	2	80.23	< 0.001	Increasing	0.39	0.36	0.42
				Stable	0.29	0.27	0.30
				Declining	0.19	0.18	0.21
Molybdenum	2	0.063	0.94	Increasing	0.06	0.05	0.07
				Stable	0.05	0.02	0.09
				Declining	0.06	0.05	0.07

The discriminant function of the first linear discriminant was:

$$\begin{array}{cc} \mathrm{LD}= & \left({\mathrm{Cu}}^{*}\;0.806\right)+\left({\mathrm{Se}}^{*}\;0.396\right)+\left({\mathrm{Co}}^{*}-0.493\right)+\left({\mathrm{Zn}}^{*}-0.420\right)\\ & +\left({\mathrm{Fe}}^{*}\;1.105\right)+\left({\mathrm{Ca}}^{*}-0.438\right)+\left({\mathrm{Mn}}^{*}\;0.157\right) \end{array}$$

The discriminant function of the second linear discriminant was:

$$\begin{array}{cc} \mathrm{LD}= & \left({\mathrm{Cu}}^{*}\;0.621\right)+\left({\mathrm{Se}}^{*}\;0.715\right)+\left({\mathrm{Co}}^{*}\;0.024\right)+\left({\mathrm{Zn}}^{*}\;0\right)\\ & +\left({\mathrm{Fe}}^{*}-0.968\right)+\left({\mathrm{Ca}}^{*}\;0.451\right)+\left({\mathrm{Mn}}^{*}\;0.251\right) \end{array}$$

**FIGURE 4:**
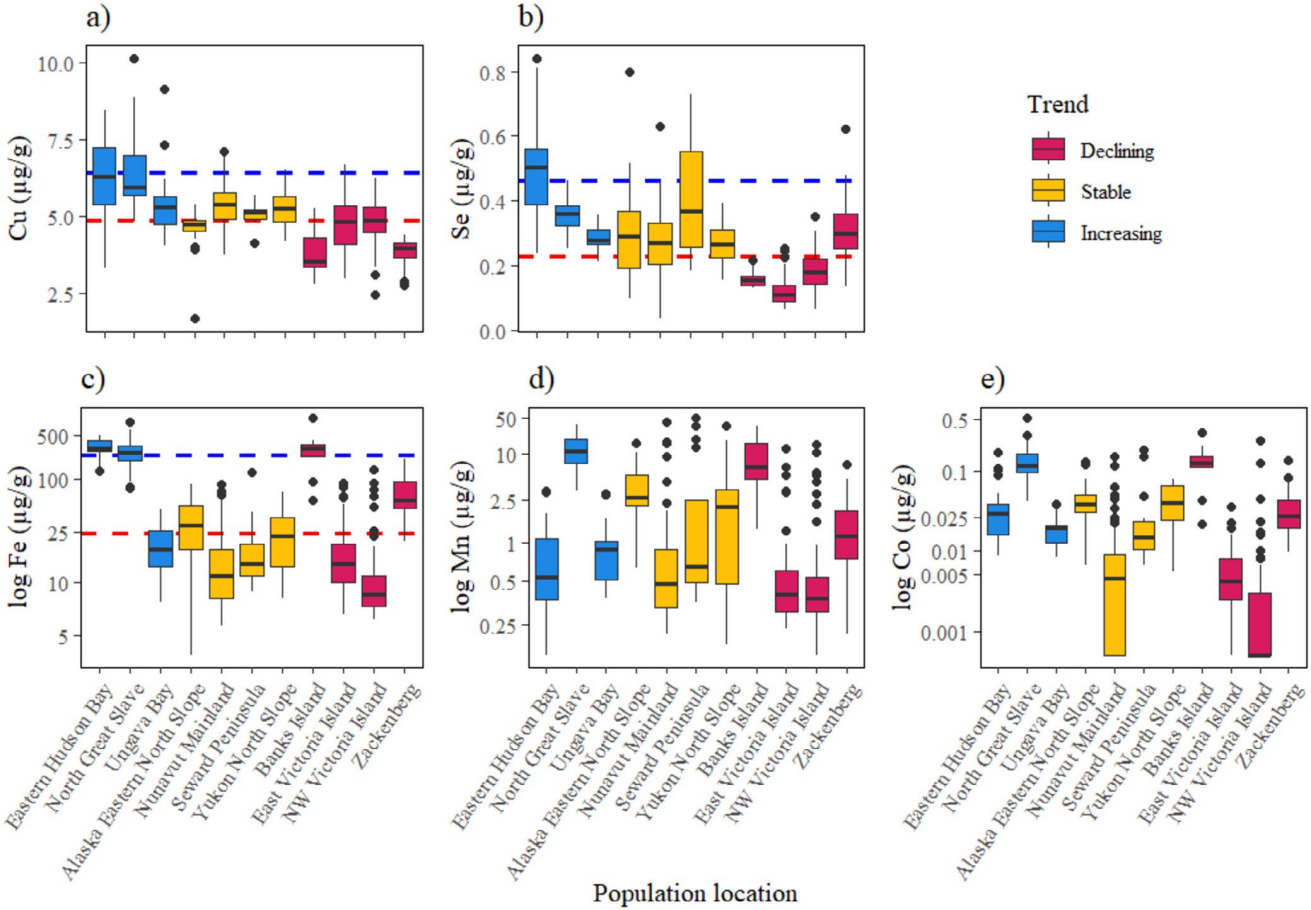
The median concentration, interquartile range, range and outliers of qiviut elements for each population measured across different qiviut growth years, grouped by population trend, for the elements which were positively associated with population trend: (a) copper (Cu), (b) selenium (Se), (c) iron (Fe), (d) manganese (Mn) and (e) cobalt (Co). The blue line shows the ‘target’ benchmark for each element and the red line shows the ‘limit’ benchmark for each element, with a probability of 0.45.

**FIGURE 5:**
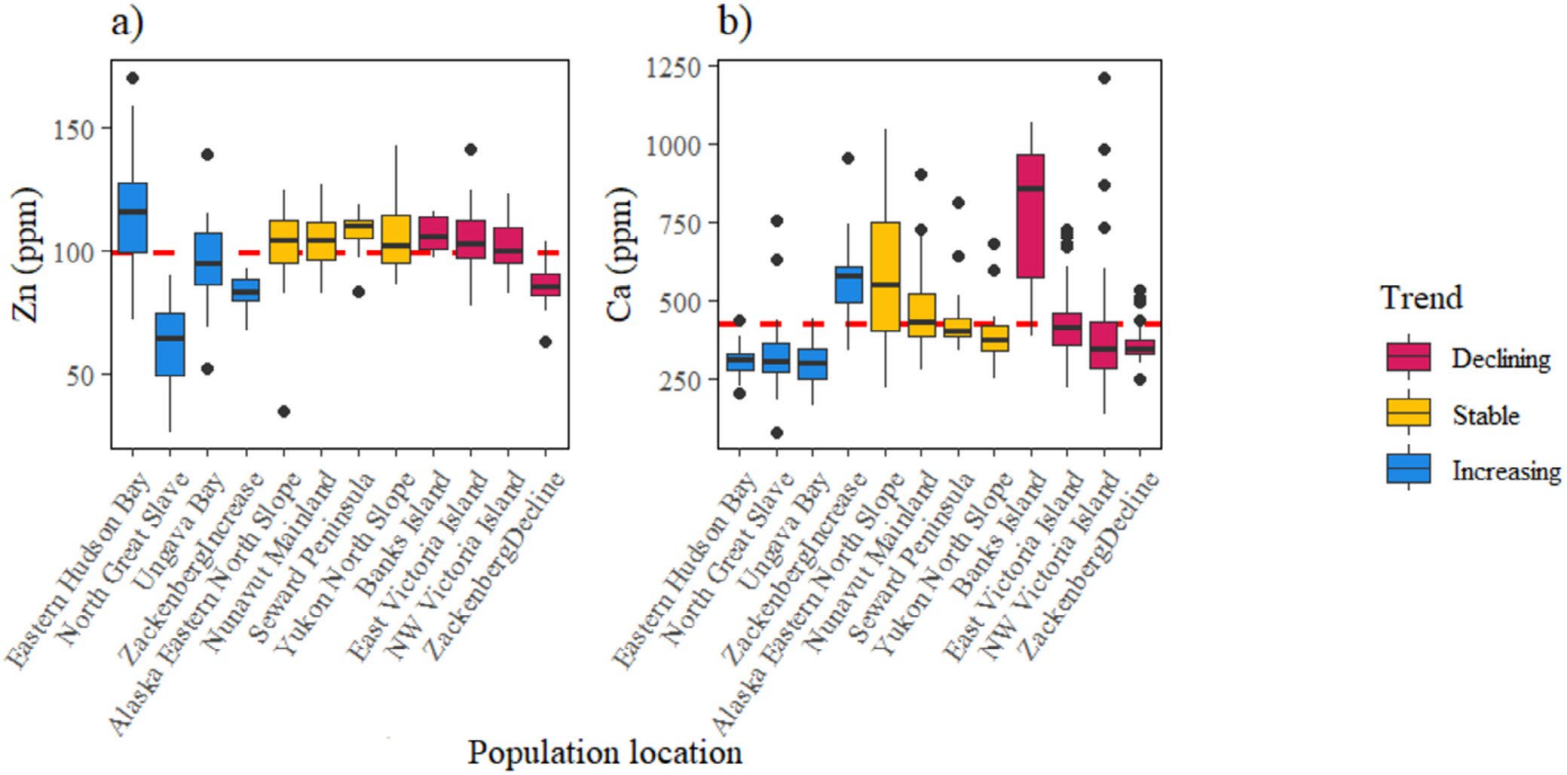
The median concentration, interquartile range, range and outliers of qiviut elements for each population measured across different qiviut growth years, grouped by population trend, for the elements which were negatively associated with population trend; (a) zinc (Zn) and (b) calcium (Ca). The red line shows the ‘limit’ benchmark above which each element has a 0.45 probability of decline.

**FIGURE 6:**
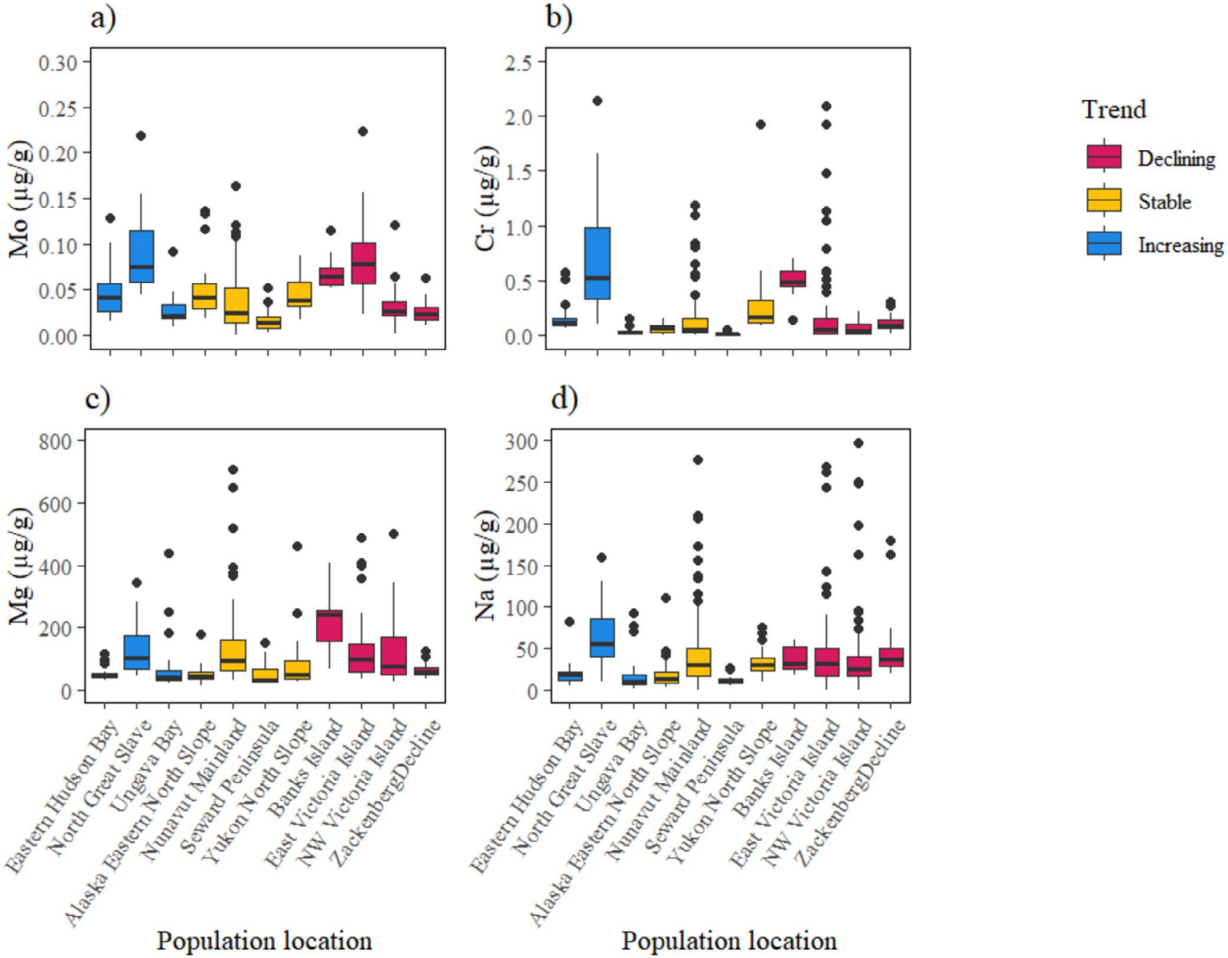
The median concentration, interquartile range, range and outliers of qiviut elements for each population measured across different qiviut growth years, grouped by population trend, for the elements which were not associated with population trend: (a) molybdenum (Mo), (b) chromium (Cr), (c) magnesium (Mg) and (d) sodium (Na).

All elements were retained in the ordinal logistic regression except for Mn (*χ* ^2^ = 3.57, *p* = 0.059) and Co (*χ* ^2^ = 1.34, *p* = 0.25; Table 3). For Cu, Se and Fe, the probability of declining populations decreased with lower element concentrations, and the probability of increasing populations increased with higher element concentrations (Figure 4). The probability of stable populations was highest at middle values of these element concentrations, and the benchmarks were defined where the probability of either an increasing or declining population was higher than the probability of a stable population (Table 3). Mn and Co did explain population trends, but benchmarks could not be estimated (Figure 4). Conversely, for Zn, populations were most likely increasing at low element concentrations (Figure 5a). For Ca, the probability of stable populations was highest at low element concentration, and the probability of declining populations increased as Ca concentrations increased (Figure 5b). Only the ‘limit’ benchmark could be defined for Ca and Zn, where populations had a higher probability of decline with values above the benchmark. Cr, Mg, Mo and Na were not associated with population trends using the ordinal logistic regression analysis (Table 3; Figure 6).

**TABLE 3:** The ordinal logistic regression (OLR) with the likelihood of the population trend changing with each element (odds ratio), including the 95% confidence interval (CI), and the ‘limit’ and ‘target’ benchmark (BM) for each element.

Variable	OLR (*t*‐value)	Odds ratio	95% CI		‘Limit’ BM (μg/g)	‘Target’ BM (μg/g)
Copper	9.35, *p* < 0.001	8.32	5.47	13.14	4.87	6.42
Selenium	7.82, *p* < 0.001	3.07	2.33	4.10	0.23	0.46
Iron	3.67, *p* < 0.001	2.09	1.53	2.98	24.66	230.50
Zinc	−4.19, *p* < 0.001	0.41	0.27	0.60	94.06	
Calcium	−2.66, *p* = 0.006	0.71	0.54	0.91	434.77	
Manganese	1.87, *p* = 0.06					
Cobalt	−1.15, *p* = 0.25					

## 4 Discussion

Element concentrations in hair have been used as a bio‐monitoring tool in a range of species, for example, antelope *Taurotragus derbianus derbianus* in Africa (Stoklasová et al. 2020), moose and caribou in North America (O'Hara et al. 2001; Jutha et al. 2022), and roe deer *Capreolus capreolus* , red deer *Cervus elaphus* and wild boar *Sus scrofa* in Europe (Montillo et al. 2019; Oropesa et al. 2022; Herrada et al. 2024), and have significant implications for ungulate conservation (Flueck et al. 2012). We evaluated qiviut trace and macro element status across 11 populations of muskoxen across the North American Arctic and found high variability in the concentration of elements in qiviut among populations. The concentration of six trace elements, Cu, Se, Fe, Mn, Co and Zn, and one macro element, Ca, was associated with population trend. We established ‘limit’ and ‘target’ benchmarks for three of these elements, Cu, Se and Fe, and ‘limit’ benchmarks for an additional two elements, Zn and Ca, which can inform population trajectory. The data presented here can be directly used as a management tool for assessing the status of muskoxen populations, and these methods can be used to support the management of other wild ungulate species.

Trace elements tended to be more similar among declining and stable populations compared to increasing populations, which were generally associated with higher concentrations of Cu, Se and Fe. Although studies most often evaluate the role of element deficiencies (Hidiroglou 1979; Flynn et al. 1977; Grace and Wilson 2002; Flueck et al. 2012), adequate concentrations of these elements have been associated with higher reproductive rates, including pregnancy (e.g., moose; Newby and DeCesare 2020), juvenile recruitment (e.g., black‐tailed deer *Odocoileus hemionus columbianus* ; Flueck 1994) and juvenile growth (e.g., red deer; Handeland et al. 2008). Our findings suggest that these elements may be more likely to facilitate population growth than to contribute to population decline. This is likely due to the effects of adequate element concentrations on reproductive success and capacity, which influence ungulate population growth (DeCesare et al. 2012). In muskoxen, Cu is particularly important for juvenile recruitment because foetuses require adequate Cu during late gestation (Rombach et al. 2003), which may be limited by maternal diet during late winter (Schmidt et al. 2023).

Mn and Co tended to have a positive association with population trend, while Zn and Ca generally were negatively associated with population trend. However, the association between these elements and population trends was small. Mn is generally maintained at stable blood concentrations unless it is deficient or below a threshold, and Co is often found at low concentrations in hair samples, which may explain why only a small effect was detected (Puls 1994; Aschner and Aschner 2005; Ceacero et al. 2009; Jutha et al. 2022). Observed deficiencies in these elements may not be directly linked to population declines. However, adequate concentrations of these elements, in conjunction with others, are likely to support successful reproduction and consequently population growth. Conversely, lower concentrations of Zn and Ca were observed in increasing populations. This may reflect the increased use of these elements resulting in lower element concentrations in the plasma and thus reduced deposition in hair (Sharma et al. 2007). Additionally, different levels of Ca intake, depending on the bioavailability of these elements in the environment, can impact observed element concentration (Littledike and Goff 1987). Interactions with other elements may also occur; for example, elevated concentrations of Zn and Ca may be linked to Cu deficiency because these elements may limit the absorption of Cu (Osredkar and Sustar 2011; Herdt and Hoff 2011), and Ca may increase when phosphorus is low in forage (Littledike and Goff 1987). In fact, a common trace element imbalance in humans is elevated Cu and low Zn, and their ratio is clinically important (Osredkar and Sustar 2011). Our understanding of element interactions in biological systems is still limited, and inferences can often be drawn from the human health literature (García‐Barrera et al. 2012).

Animal nutrition is complex, and the extent to which other elements are present can influence interactions (Soetan et al. 2010). Adequate nutritional intake of all essential elements is most likely to facilitate healthy wildlife populations and successful reproduction (National Research Council 2007; Soetan et al. 2010). Additionally, synergistic or antagonistic interactions occur at deficient and excess concentrations of elements; thus, elements cannot be considered in isolation (García‐Barrera et al. 2012). In the present study, elements that contributed to the discriminatory analysis, ordered by their discriminant function coefficient, were Cu, Se, Fe, Mn, Co, Zn and Ca. While these elements together explained some of the variation in population trend, only three, Cu, Se and Fe, could be used to define ‘limit’ and ‘target’ benchmarks. Dietary intake has been associated with hair concentrations of Cu, Se, Mo and Co (Combs et al. 1982; Cunningham and Hogan 1958; Ghorbani et al. 2015; Kellaway et al. 1978). In contrast, the status of elements such as Fe and Zn may not be well represented by hair (Combs et al. 1982; Roug et al. 2015). While the benchmark values are useful for assessing population status, the overall hair element profile should be referred to for a broader understanding of nutritional status.

The concentrations of Cr, Na, Mg and Mo did not reflect muskox population trends in this study, suggesting that the nutritional significance of these elements was not adequately reflected in hair samples. The mechanisms through which elements are metabolised in the body, deposited in growing hair and their interactions may complicate how these concentrations are measured. This is due to metabolic interactions and fluctuations in serum and organ levels, as well as bioavailability (Combs 1987). Thus, hair element concentrations and their relevance to individual or population health may be more complicated and not adequately reflected in this tissue. Mo is regularly included in studies assessing the role of elements on ungulate populations, but Cr, Na and Mg are not frequently considered (Webster et al. 2021; Jutha et al. 2022; Mosbacher et al. 2022; van Beest et al. 2023) except see Forchhammer and Boomsma (1995) for the uptake of Na during winter foraging strategies in muskoxen. These elements are relevant for ungulate health, including growth, reproduction and survival, but have not yet been associated with population‐level effects (Ropstad et al. 1997; Staaland and White 2001; Rioux et al. 2022). Although Rioux et al. (2022) suggested that adult caribou survival is linked to higher concentrations of Na, the reported confidence interval includes zero, which leaves uncertainty about the presence of an effect.

We found high variation in qiviut trace element profiles within and between muskox populations. Confounding factors such as landscape geochemistry, season, nutrition, animal age, and body position of hair may influence hair trace element concentrations (Combs 1987; Roug et al. 2015; Di Francesco et al. 2022). While there were some significant effects of age, sex and collection type, most of the variation was accounted for by interpopulation and interannual differences. This variation may be driven by environmental changes, such as latitudinal variation in the bioavailability of elements, or annual changes in environmental drivers of bioavailability, such as weather patterns. The large geographical scale of this study means that habitat, and consequently bioavailability, is likely to vary among regions. Exploring the drivers of this variation, understanding region or habitat‐specific differences and how this may impact muskox populations is an important avenue of research. Despite this potential for confounding factors, we were able to show the associations between qiviut element status and population trends across a large geographical scale. A small effect of collection type was identified for Mn and Co, where element concentration in ground collected samples was higher, and Se, where element concentration was lower in harvest samples. Appropriate care must be taken when collecting samples using different methods and when interpreting results (Rakic et al. 2023). Although we did not find consistent differences in element concentrations depending on collection method, we recommend sample collection be conducted consistently to reduce potential variation. Ultimately, more detailed knowledge about element levels, particularly in hair, and how these are correlated with field and clinical observations including the biochemical status of vegetation (Oster et al. 2018; van Beest et al. 2023), will improve inferences that can be made about trace element concentrations in ungulates.

In this study, we showed that hair concentrations of certain elements may reflect different population trends in muskoxen, and we have identified threshold values for elements associated with increasing, stable and declining populations. These findings were also supported by Mosbacher et al. (2022) which found similar thresholds for Cu and Se in qiviut samples collected from the ground. Identifying threshold values that can provide insights into the status of wildlife populations can be an invaluable and actionable tool for wildlife managers (Peacock et al. 2020; White et al. 2014). Benchmarks can be used to define whether a population is healthy and resilient (‘target’) or at risk of extirpation due to decline (‘limit’; Peacock et al. 2022). Establishing these benchmarks requires defining how changes in metrics, which are sensitive and specific to population health, correspond to changes in population health (Rice and Rochet 2005). This can be accomplished by collecting information over an appropriate temporal and spatial range using standardised approaches (Kutz et al. 2013). By incorporating this non‐invasive bio‐monitoring tool into muskox population management on an annual basis alongside population surveys, a comprehensive longitudinal database can be established to track changes in population trajectory and hair element status. Region‐specific differences may also be important for population health; thus, longitudinal data can provide information on the specificity of benchmarks to regions. While Cu, Se and Fe were the most useful elements for defining benchmarks, it is important to recognise the broader element profile reflected in hair, including Co, Zn, Mn and Ca, which collectively play a role in ungulate health and reproduction. When using these benchmarks, it should be noted that in this study we found that higher concentrations of elements were generally beneficial for muskox populations; however, toxicity can occur when trace element concentrations are in excess (Gamberg et al. 2016; Durkalec et al. 2018). Incorporating routine screening for element concentrations, as demonstrated in this study with muskoxen, can support wildlife population monitoring and management practices. Qiviut and hair from other ungulates are easy to collect passively from the environment or through anyone with access to animals through harvesting or monitoring practices and can be simply stored at room temperature (Combs 1987; Jutha et al. 2022). Using benchmarks from individual elements or trace and macro element profiles measured from hair can provide a timely and effective tool to assess population status, thereby supporting proactive wildlife management and conservation.

## Author Contributions

**Eleanor R. Dickinson:** data curation (equal), formal analysis (lead), visualization (lead), writing – original draft (lead), writing – review and editing (lead). **Jesper Bruun Mosbacher:** conceptualization (equal), data curation (lead), methodology (equal), visualization (equal), writing – review and editing (equal). **Colleen Arnison:** data curation (equal), methodology (equal), resources (equal), writing – review and editing (equal). **Kimberlee Beckmen:** data curation (equal), methodology (equal), writing – review and editing (equal). **Steeve D. Côté:** conceptualization (equal), data curation (equal), methodology (equal), writing – original draft (equal), writing – review and editing (equal). **Juliette Di Francesco:** data curation (equal), methodology (equal), writing – review and editing (equal). **Sophia V. Hansson:** data curation (equal), methodology (equal), resources (equal), writing – review and editing (equal). **Elham Z. Jahromi:** data curation (equal), methodology (equal), resources (equal), writing – review and editing (equal). **David W. Kinniburgh:** data curation (equal), methodology (equal), resources (equal), writing – review and editing (equal). **Gäel Le Roux:** data curation (equal), resources (equal), writing – review and editing (equal). **Lisa‐Marie Leclerc:** data curation (equal), methodology (equal), writing – review and editing (equal). **Fabien Mavrot:** data curation (equal), methodology (equal), writing – review and editing (equal). **Niels M. Schmidt:** data curation (equal), methodology (equal), writing – review and editing (equal). **Michael J. Suitor:** data curation (equal), methodology (equal), writing – review and editing (equal). **Joëlle Taillon:** data curation (equal), methodology (equal), writing – review and editing (equal). **Matilde Tomaselli:** data curation (equal), methodology (equal), writing – review and editing (equal). **Susan J. Kutz:** conceptualization (equal), data curation (equal), methodology (equal), visualization (equal), writing – original draft (equal), writing – review and editing (equal).

## Conflicts of Interest

The authors declare no conflicts of interest.

## Statement on Inclusion

Our study brings together authors based in the countries where the study was carried out. All authors were engaged early on with the research and study design to ensure that the diverse sets of perspectives they represent were considered from the onset. The study builds on local management plans and published literature, as well as the knowledge and perspectives of Indigenous communities. Results of this study have been communicated to Indigenous communities and local wildlife co‐management organisations.

## Supporting information


Appendix S1.

## Data Availability

Data and code are available to access on GitHub by following https://github.com/ERDickinson0/muskox_elements.
