# Supplementary material for: Qiviut Trace and Macro Element Profile Reflects Muskox Population Trends
Source: Ecol Evol. 2025 Feb 20;15(2):e71020. doi: 10.1002/ece3.71020 (PMC11842511; doi:10.1002/ece3.71020)
Supplement: Supplementary file 1 — Appendix S1. [file ECE3-15-e71020-s001.docx]

**Supporting Information**

**Appendix S1: Materials and Methods**

**Ethics statement**

Samples were obtained under the relevant animal care and wildlife research permits, for captures (Alaska Department of Fish and Game IACUC #06-08, #0075-2018-69; Canada NWT WRP #WL5004469, 500664, GN WRP #WL2016-058; Greenland G13-029, G15-019, G17-014; University of Calgary Animal Care Committee AC22-0060). No animals were killed or captured specifically for this study.

**Qiviut processing and analyses**

All samples were processed at the Alberta Centre for Toxicology, University of Calgary, Calgary, Canada, except for the samples collected from the Eastern Hudson Bay, Yukon North Slope (captures only), and Zackenberg populations. These samples were processed at the Observatoire Midi-Pyrénées (OMP), Centre de Recherche sur la Biodiversité et l'Environnement (CRBE) / Centre National de la Recherche Scientifique (CNRS), Toulouse, France. For each sample, guard hairs and large external contaminants, such as soil and vegetation, were removed from the qiviut using plastic tweezers. The samples were washed twice in 95% ethanol and Milli-Q Type 1 Ultrapure water (mQ H_2_O) to remove further contamination, before being stored in clean paper bags and oven-dried at 50 °C for 48 hours. Samples were analysed using either inductively coupled plasma mass spectrometry (ICP-MS; 8800 Triple Quadrupole ICP-MS, Agilent) or a combination of high-resolution inductively coupled plasma mass spectrometry (HR-ICP-MS) and ICP-OES (as described in Mosbacher et al., 2022).

**Qiviut analyses: Alberta Centre for Toxicology**

For each sample, approximately 30 to 50 mg (mean ± SD = 41.28 ± 8.26 mg) of the dried qiviut sample was accurately weighed into a Teflon vial with 2 ml concentrated nitric acid (70% HNO_3_, TraceMetalTM Grade, Thermo Fisher Scientific). Samples were digested using a microwave digestion system (ETHOS EZ Microwave Digestion System and microsampling inserts; Milestone, Sorisole, Italy) by gradually increasing temperature to 180 °C over 25 minutes at 1000 W, and then keeping the temperature at 180 °C for 15 minutes. After digestion and cooling, each sample was diluted with ultrapure water to a final volume of 4 ml. Further dilution 1:10 with ultrapure water was performed before analysing samples. The qiviut concentrations of 11 elements (Cu, Se, Co, Zn, Mn, Mg, Na, Fe, Ca, Mo, and Cr) were determined using inductively coupled plasma mass spectrometry (ICP-MS; 8800 Triple Quadrupole ICP-MS, Agilent). Each batch of samples included qiviut samples, certified reference materials (NIST 2976 freeze-dried mussel tissue and DORM-3 fish protein) for quality control, and a blank sample as negative control (ultrapure water and HNO_3_).

**Qiviut analyses: Observatoire Midi-Pyrénées**

For each sample, approximately 134 to 160 mg (mean ± SD = 149.50 ± 8.80 mg) of qiviut was placed in acid cleaned digitubes (SCP Sciences 010-500-263) together with 3 ml of ultrapure nitric acid (HNO_3_; ~67-69%, OptimaTM Grade, Fisher) closed with airtight caps, and digested overnight at 90 °C. After being cooled down to room temperature the digestions were diluted in two steps; first the digest was transferred to 15 mL Falcon tubes and diluted with mQ H_2_O (3:7 digest:mQ H_2_O) to create a solution which was stored in dark conditions at ~6°C until further processing. Two subsamples of this solution was taken, and each sample was further diluted to 10ml with mQ H_2_O. These were then analysed with a High Resolution Inductively Coupled Plasma Mass Spectrometer (HR-ICP-MS; Thermo-Fisher Element-XR) for Cu, Se, Mo, Co, Cr and Zn at the OMP, and Inductively Coupled Plasma Optical Emission Spectrometer (ICP-OES, IRIS Intrepid II Thermo Electron for Na, Mn, Mg, Fe, and Ca at the CRBE/CNRS EcoLab. Each batch of samples included qiviut samples, certified reference materials, and a blank sample as negative control (ultrapure water and HNO_3_). Certified reference materials were used to check the accuracy of the digestion and analysis protocol; PRON-1 (River prawn/Crevette, NRC Canada), IAEA-336 (Lichen) and NIST 1515 (Apple leaves).

**Appendix S2: Results**

**Table S1:** Reported limit of quantitation (LOQ), the percentage of observations that were below the LOQ, the mean % difference between duplicates with the standard deviation (SD), the percent of duplicates that were more than 20% different, and the mean concentration of blank reference samples with the SD, for the samples analysed at the Alberta Centre for Toxicology, University of Calgary, Calgary, Canada.

| **Element** | **Method LOQ (mg/L)** | **% <LOQ** | **Duplicate mean % difference (± SD)** | **% difference more than ±20%** | **Blank mean concentration (± SD)** | **DORM-3 reference target value (± SD)** | **DORM-3 reference mean result (± SD)** | **NIST 2976 reference target value (± SD)** | **NIST 2976 reference mean result (± SD)** |
| --- | --- | --- | --- | --- | --- | --- | --- | --- | --- |
| Na | 0.3 | 46 | 4.98 (± 14.42) | 3.70 | 0.03 (± 0.11) |  |  | 35000 (± 7000) | 34951.05 (± 1562.89) |
| Mg | 0.1 | 0 | 2.20 (± 3.17) | 0.00 | 0.01 (± 0.02) |  |  | 5300 (± 1060) | 5080.10 (± 246.26) |
| K | 0.1 | 0 | 4.56 (± 7.39) | 3.70 | 0.11 (± 0.17) |  |  | 9700 (± 1940) | 10485.08 (± 363.25) |
| Ca | 0.1 | 0 | 1.65 (± 2.56) | 0.00 | 0.28 (± 0.91) |  |  | 7600 (± 1520) | 7439.69 (± 338.44) |
| Cr | 0.0005 | 36 | 4.61 (± 13.74) | 25.93 | 0.00 (± 0.01) | 1.89 (± 0.38) | 1.84 (± 0.17) | 0.50 (± 0.10) | 0.50 (± 0.04) |
| Mn | 0.0001 | 0 | 2.14 (± 3.73) | 3.70 | 0.00 (± 0.00) |  |  | 33.00 (± 6.60) | 40.63 (± 2.00) |
| Fe | 0.005 | 0 | 2.37 (± 3.46) | 0.00 | 0.02 (± 0.06) | 347.00 (± 69.40) | 371.92 (± 38.02) | 171.00 (± 34.20) | 185.49 (± 13.60) |
| Co | 0.00005 | 18 | 3.33 (± 14.56) | 14.81 | 0.00 (± 0.00) |  |  | 0.61 (± 0.12) | 0.66 (± 0.04) |
| Cu | 0.0005 | 0 | 1.40 (± 1.90) | 0.00 | 0.02 (± 0.11) | 15.50 (± 3.10) | 16.66 (± 1.21) | 4.02 (± 0.80) | 14.78 (± 25.17) |
| Zn | 0.0005 | 0 | 1.13 (± 1.40) | 0.00 | 0.05 (± 0.21) | 51.30 (± 10.26) | 53.60 (± 3.04) | 137.00 (± 27.40) | 175.65 (± 45.40) |
| As | 0.0005 | 8 | 1.30 (± 4.56) | 7.41 | 0.00 (± 0.00) | 6.88 (± 1.38) | 6.60 (± 0.43) | 13.30 (± 2.66) | 14.18 (± 1.17) |
| Se | 0.0001 | 0 | 3.9 (± 6.37) | 7.41 | 0.00 (± 0.00) |  |  | 1.80 (± 0.36) | 1.96 (± 0.19) |
| Mo | 0.00005 | 4 | 4.57 (± 15.30) | 29.63 | 0.00 (± 0.00) |  |  |  |  |
| Cd | 0.0005 | 36 | 5.20 (± 20.81) | 33.33 | 0.00 (± 0.00) | 0.29 (± 0.06) | 0.30 (± 0.05) | 0.82 (± 0.16) | 0.89 (± 0.08) |
| Pb | 0.00005 | 3 | 2.25 (± 5.19) | 22.22 | 0.00 (± 0.01) | 0.40 (± 0.08) | 0.39 (± 0.03) | 1.19 (± 0.24) | 2.47 (± 2.85) |

**Table S2:** Reported the mean concentration of blank reference samples with the SD, for the samples analysed at the Observatoire Midi-Pyrénées, Toulouse, France. The accuracy of digestion and analysis protocol was checked using certified standards; PRON-1 (River prawn/Crevette, NRC Canada), IAEA-336 (Lichen) and NIST 1515 (Apple leaves). For all samples, blanks were negligible, and the concentrations measured were within the certified values for all trace elements. No samples were below limits of detection.

| **Element** | **Machine** | **IAEA-336 reference target value (range)** | **IAEA-336 reference mean result (± SD) n = 4** | **NIST 1515 reference target value (± SD)** | **NIST 1515 reference mean result (± SD) n = 6** | **PRON-1 reference target value (± 20%)** | **PRON-1 reference mean result (± SD) n = 6** |
| --- | --- | --- | --- | --- | --- | --- | --- |
| Na | ICP-OES | 320 (280 - 360) | 267.33 ± 6.06 | 24.4 ± 1.2 | 25.72 ± 1.47 | 4080 ± 440 | 4228.28 ± 114.51 |
| Mg | ICP-OES |  | 497.1 ± 13.06 | 2710 ± 80 | 2545.58 ± 67.36 | 1320 ± 140 | 1284.8 ± 45.87 |
| K | ICP-OES | 1840 (1640 - 2040) | 1559.81 ± 33.84 | 1610 ± 200 |  | 11600 ± 3600 | 11773.7 ± 455.46 |
| Ca | ICP-OES |  | 2330.32 ± 90.43 | 1526 ± 150 |  | 1450 ± 160 | 1394.49 ± 75.03 |
| Cr | HR-ICP-MS |  | 0.69 ± 0.34 |  | 0.19 ± 0.13 |  | 1.45 ± 0.45 |
| Mn | ICP-OES | 63 (56 - 70) | 57.71 ± 1.75 | 54 ± 3 | 48.19 ± 1.52 | 4.6 ± 0.8 | 4.6 ± 0.2 |
| Fe | ICP-OES | 430 (380 - 480) | 331.47 ± 9.8 | 55.77 ± 2.01 | 16 | 12.54 ± 1 | Fe |
| Co | HR-ICP-MS | 0.29 (0.24 - 0.34) | 0.28 ± 0.06 | 0.09 | 0.08 ± 0.03 | 0.06 | 0.04 ± 0.01 |
| Cu | HR-ICP-MS | 3.6 (3.1 - 4.1) | 3.48 ± 0.53 | 5.64 ± 0.24 | 5.2 ± 1.44 | 23.2 ± 2.0 | 21.79 ± 3.31 |
| Zn | HR-ICP-MS | 30.4 (27.0 - 33.8) | 28.46 ± 1.93 | 12.5 ± 0.3 | 10.26 ± 2.26 | 58.0 ± 8.0 | 50.27 ± 5.9 |
| As | HR-ICP-MS | 0.63 (0.55 - 0.71) | 0.565 ± 0.072 | 0.038 ± 0.007 | 0.031 ± 0.007 | 1.73 ± 0.12 | 1.51 ± 0.203 |
| Se | HR-ICP-MS |  | 0.23 ± 0.02 | 0.05 ± 0.01 | 0.04 ± 0.02 | 0.97 ± 0.14 | 1.04 ± 0.14 |
| Mo | HR-ICP-MS |  | 0.06 ± 0.02 | 0.09 ± 0.01 | 0.12 ± 0.05 |  | 0.05 ± 0.02 |
| Cd | HR-ICP-MS | 0.12 | 0.11 ± 0.03 | 0.01 | 0.01 ± 0 | 0.02 ± 0.01 | 0.01 ± 0 |
| Pb | HR-ICP-MS | 4.9 (4.3 - 5.5) | 4.55 ± 0.49 | 0.47 ± 0.02 | 0.36 ± 0.07 | 0.04 ± 0.01 | 0.04 ± 0.01 |

**Table S3:** Overview of populations assessed in this study ranging from Alaska to Greenland with current population trends and sizes, and the median concentrations with the first and third quantile of elements measured in qiviut. The hair growth year refers to the year of hair formation, rather than year of collection.

| **Population** | **Hair Growth Year(s)** | **N** | **Collection method** | **Population Trend** | **Median element concentration (first and third quantiles; µg/g)** | | | | | | | | | | |
| --- | --- | --- | --- | --- | --- | --- | --- | --- | --- | --- | --- | --- | --- | --- | --- |
|  |  |  |  |  | **Na** | **Mg** | **Ca** | **Cr** | **Mn** | **Fe** | **Co** | **Cu** | **Zn** | **Se** | **Mo** |
| Alaska Eastern North Slope | 2006-2007 | 18 | Capture | Stable | 12.94 (8.96, 22.09) | 45.06 (37.69, 58.77) | 548.17 (405.94, 750.74) | 0.06 (0.03, 0.08) | 2.67 (2.17, 4.88) | 29.10 (17.84, 47.94) | 0.04 (0.03, 0.050) | 4.72 (4.51, 4.87) | 104.50 (94.99, 112.25) | 0.29 (0.19, 0.37) | 0.04 (0.03, 0.06) |
| Seward Peninsula | 2008, 2018 | 11 | Capture | Stable | 9.86 (9.13, 13.43) | 29.48 (27.65, 65.95) | 403.59 (387.98, 442.70) | 0.01 (0.01, 0.02) | 0.65 (0.50, 4.02) | 13.59 (11.04, 19.77) | 0.01 (0.01, 0.02) | 5.11 (4.89, 5.23) | 109.83 (105.09, 112.22) | 0.37 (0.26, 0.55) | 0.01 (0.01, 0.02) |
| Yukon North slope | 2016-2018 | 20 | Capture (n = 14); Shed (n = 6) | Increasing | 30.04 (23.72, 37.81) | 43.40 (36.67, 66.80) | 374.94 (338.48, 421.09) | 0.16 (0.12, 0.29) | 2.12 (0.48, 3.26) | 23.08 (13.26, 34.73) | 0.04 (0.02, 0.06) | 5.24 (4.82, 5.63) | 102.25 (94.84, 114.27) | 0.27 (0.23, 0.31) | 0.04 (0.03, 0.06) |
| Banks Island | 2017 | 9 | Shed | Declining | 31.38 (24.83, 52.82) | 242.88 (154.76, 257.02) | 858.66 (572.38, 964.65) | 0.49 (0.45, 0.59) | 6.33 (4.31, 14.60) | 283.27 (219.84, 331.67) | 0.12 (0.11, 0.15) | 3.52 (3.35, 4.32) | 105.82 (100.60, 113.90) | 0.15 (0.14, 0.17) | 0.06 (0.06, 0.07) |
| NW Victoria Island | 2016-2018 | 44 | Hunted | Declining | 24.68 (17.52, 57.79) | 74.65 (45.64, 172.72) | 349.11 (286.60, 431.71) | 0.04 (0.02, 0.11) | 0.38 (0.31, 0.54) | 8.34 (7.10, 11.15) | 0.00 (0.00, 0.00) | 4.86 (4.46, 5.29) | 100.19 (94.92, 109.22) | 0.18 (0.14, 0.22) | 0.03 (0.02, 0.04) |
| North Great Slave | 2017 | 22 | Shed | Increasing | 51.97 (38.97, 81.41) | 97.03 (69.05, 130.27) | 307.44 (271.14, 361.48) | 0.45 (0.31, 0.83) | 10.73 (7.15, 17.28) | 249.8 (192.96, 316.76) | 0.11 (0.09, 0.17) | 5.93 (5.68, 6.96) | 64.29 (49.40, 74.24) | 0.36 (0.32, 0.39) | 0.08 (0.06, 0.11) |
| East Victoria Island (MX-07) | 2014-2018 | 58 | Hunted | Declining | 31.14 (15.72, 51.72) | 100.45 (59.45, 147.70) | 415.38 (359.67, 462.68) | 0.07 (0.02, 0.46) | 0.41 (0.31, 0.60) | 13.51 (9.85, 19.77) | 0.00 (0.00, 0.01) | 4.80 (4.07, 5.33) | 102.80 (97.21, 112.52) | 0.11 (0.09, 0.14) | 0.08 (0.06, 0.12) |
| Nunavut Mainland (MX-09) | 2013-2018 | 107 | Hunted | Stable | 31.62 (17.97, 53.82) | 95.46 (63.48, 165.19) | 434.58 (388.04, 520.33) | 0.06 (0.03, 0.16) | 0.48 (0.33, 0.89) | 11.06 (7.87, 17.84) | 0.00 (0.00, 0.01) | 5.36 (4.92, 5.76) | 104.10 (96.63, 111.44) | 0.27 (0.02, 0.33) | 0.02 (0.01, 0.05) |
| Eastern Hudson Bay | 2016 | 30 | Capture | Increasing | 18.28 (11.68, 22.82) | 46.54 (38.4, 56.00) | 313.03 (280.14, 329.00) | 0.13 (0.09, 0.15) | 0.54 (0.37, 1.08) | 290.10 (259.59, 388.93) | 0.03 (0.02, 0.04) | 6.30 (5.37, 7.23) | 115.66 (99.50, 127.71) | 0.50 (0.39, 0.56) | 0.04 (0.03, 0.06) |
| Ungava Bay | 2018 | 19 | Capture | Increasing | 9.82 (6.34, 18.41) | 40.2 (32.51, 61.26) | 299.07 (249.79, 345.72) | 0.02 (0.01, 0.04) | 0.89 (0.51, 1.03) | 17.72 (12.79, 25.9) | 0.02 (0.01, 0.02) | 5.28 (4.73, 5.65) | 94.69 (86.36, 106.94) | 0.28 (0.27, 0.31) | 0.02 (0.02, 0.03) |
| Zackenberg | 2013, 2015, 2017 | 44 | Capture | Declining | 37.05 (28.81, 50.66) | 58.62 (49.62, 70.88) | 344.00 (330.22, 373.47) | 0.09 (0.07, 0.13) | 1.15 (0.74, 1.95) | 55.08 (43.70, 93.46) | 0.03 (0.02, 0.04) | 3.94 (3.65, 4.13) | 85.49 (81.73, 90.81) | 0.30 (0.25, 0.36) | 0.02 (0.02, 0.03) |

**Table S4:** The effect of collection type (harvest, capture or ground collection) on the amount of element measured in muskox qiviut. ΔAIC to the intercept model. Marginal R-squared (R^2^m) includes the variation explained by retained variables in the top model, and conditional R-squared (R^2^c) includes the variation explained by retained variables and the nested random effects of population and year.

| **Model** | **Collection type** | | **df** | **ΔAIC_c_** | **R^2^m** | **R^2^c** |
| --- | --- | --- | --- | --- | --- | --- |
|  | **LRT** | **p-value** |  |  |  |  |
| Sodium (logNa+1) | 7.24 | 0.007** | 5 | 3.71 | 0.046 | 0.094 |
| Magnesium (logMg) | 6.92 | 0.0085** | 5 | 12.32 | 0.066 | 0.16 |
| Calcium (logCa) | 1.39 | 0.24 | 5 | 3.23 | 0.022 | 0.24 |
| Chromium (sqrtCr) | 2.28 | 0.13 | 5 | 1.86 | 0.018 | 0.078 |
| Manganese (logMn) | 12.64 | <0.001*** | 5 | 7.03 | 0.16 | 0.22 |
| Iron (logFe) | 4.59 | 0.032* | 5 | 0.52 | 0.059 | 0.21 |
| Cobalt (logCo) | 7.82 | 0.0052** | 5 | 5.47 | 0.17 | 0.38 |
| Copper (Cu) | 1.45 | 0.48 | 5 | 2.70 | 0.013 | 0.52 |
| Zinc (Zn) | 0.35 | 0.55 | 5 | 1.83 | 0.0022 | 0.038 |
| Selenium (logSe) | 8.22 | 0.016* | 5 | 0.52 | 0.28 | 0.64 |
| Molybdenum (logMo) | 8.23 | 0.016* | 5 | 2.52 | 0.052 | 0.36 |

**Table S5:** The effect of animal age class (calf, yearling, adult), and sex (male, female) on the concentration of element measured in muskox qiviut. ΔAIC to the intercept model. Marginal R-squared (R^2^m) includes the variation explained by retained variables in the top model, and conditional R-squared (R^2^c) includes the variation explained by retained variables and the nested random effects of population and year.

| **Model** | **Age** | | **Sex** | | **df** | **R^2^m** | **R^2^c** |
| --- | --- | --- | --- | --- | --- | --- | --- |
|  | **LRT** | **p-value** | **LRT** | **p-value** |  |  |  |
| Sodium (logNa+1) | 0.41 | 0.81 | 0.17 | 0.68 | 7 | 0.0016 | 0.11 |
| Magnesium (logMg) | 2.66 | 0.26 | 3.02 | 0.082 | 7 | 0.016 | 0.32 |
| Calcium (logCa) | 1.17 | 0.56 | 0.021 | 0.88 | 7 | 0.004 | 0.23 |
| Chromium (sqrtCr) | 1.49 | 0.48 | 3.15 | 0.076 | 7 | 0.018 | 0.13 |
| Manganese (logMn) | 4.94 | 0.085 | 0.37 | 0.54 | 7 | 0.014 | 0.41 |
| Iron (logFe) | 2.32 | 0.31 | 0.76 | 0.38 | 7 | 0.010 | 0.28 |
| Cobalt (logCo) | 8.40 | 0.015* | 0.0019 | 0.96 | 7 | 0.018 | 0.51 |
| Copper (Cu) | 1.37 | 0.50 | 3.45 | 0.063 | 7 | 0.018 | 0.22 |
| Zinc (Zn) | 1.27 | 0.53 | 0.42 | 0.51 | 7 | 0.0068 | 0.037 |
| Selenium (logSe) | 13.59 | 0.0011** | 4.93 | 0.026* | 7 | 0.032 | 0.64 |
| Molybdenum (logMo) | 1.12 | 0.57 | 8.41 | 0.0037** | 7 | 0.028 | 0.40 |
